# Improving GP-CMHH Communication

**DOI:** 10.1192/bjo.2026.11397

**Published:** 2026-06-30

**Authors:** Graham Behr, Enya Aqulina, Kristy Ieong, Declan Brogan, Darryl Murray

**Affiliations:** CNWL, London, United Kingdom

## Abstract

**Aims:**

The aim of the project was to formalise and structure reciprocal communication between GPs and Queen’s Park and Paddington community mental health hub (QPP CMHH), with the hope of streamlining mental health care, supporting GP colleagues with mental health management, and to improve the quality of referrals.

**Methods:**

Three GP surgeries were selected on the basis that they constituted 80% of the referrals to QPP CMHH. A7-item questionnaire (consisting of 5 rating-scale questions, ranging from dissatisfied to very satisfied, and 2 free-text questions) was sent to the GPs at these practices. Both virtual and paper copies were used. GPs were then given access to a spreadsheet that allowed them to book in for patients that they wished to discuss with CMHH, where they provided patient details and the clinical question. Virtual meetings were held every 2 weeks to discuss the cases and for the GPs to receive advice.

**Results:**

A total of 15 pre-intervention questionnaires were returned, from 3 GP surgeries. A total of 13 post-intervention questionnaires were returned, from 1 GP surgery. There was overall improvement in the GP-CMHH relationship, with the greatest improvement seen in the domain ‘communications were being sent via the correct channels’.

**Conclusion:**

Limitations include long-term sustainability, which depends heavily on the commitment and input of more permanent, often senior staff. Suggested areas for improvement included a bypass telephone number to expedite access to QPP professionals for better support for duty GPs, and integrating the discussion referral process into SystmOne, the software that both GP surgeries and CMHT use. Suggested next steps is to widen access to additional GP surgeries.

